# I-Space: The Effects of Emotional Valence and Source of Music on Interpersonal Distance

**DOI:** 10.1371/journal.pone.0026083

**Published:** 2011-10-12

**Authors:** Ana Tajadura-Jiménez, Galini Pantelidou, Pawel Rebacz, Daniel Västfjäll, Manos Tsakiris

**Affiliations:** 1 Department of Psychology, Royal Holloway, University of London, Egham, Surrey, United Kingdom; 2 Department of Psychology, Goldsmiths, University of London, London, United Kingdom; 3 Division of Applied Acoustics, Chalmers University of Technology, Gothenburg, Sweden; University of Bologna, Italy

## Abstract

**Background:**

The ubiquitous use of personal music players in over-crowded public transport alludes to the hypothesis that apart from making the journey more pleasant, listening to music through headphones may also affect representations of our personal space, that is, the emotionally-tinged zone around the human body that people feel is “their space”. We evaluated the effects of emotional valence (positive versus negative) and source (external, i.e. loudspeakers, versus embedded, i.e. headphones) of music on the participant's interpersonal distance when interacting with others.

**Methodology/Principal Findings:**

Personal space was evaluated as the comfort interpersonal distance between participant and experimenter during both active and passive approach tasks. Our results show that, during passive approach tasks, listening to positive versus negative emotion-inducing music reduces the representation of personal space, allowing others to come closer to us. With respect to a no-music condition, an embedded source of positive emotion-inducing music reduced personal space, while an external source of negative emotion-inducing music expanded personal space.

**Conclusions/Significance:**

The results provide the first empirical evidence of the relation between induced emotional state, as a result of listening to positive music through headphones, and personal space when interacting with others. This research might help to understand the benefit that people find in using personal music players in crowded situations, such as when using the public transport in urban settings.

## Introduction

“Personal space” is defined in social psychological literature as the emotionally-tinged zone around the human body that people feel is “their space” [1], that is, the space which others cannot intrude without arousing discomfort [2]. In the field of cognitive neurosciences this region of space is sometimes referred as “peripersonal space”, and described as “the near space immediately surrounding the body” [3]. While social psychology has studied how personal space is modulated while participants interact with other people, the study of peripersonal space in experimental psychology and cognitive neurosciences has looked mainly at the differential processing of multisensory stimuli that fall within or outside this space, most often in the absence of any social interaction. The social psychology tradition suggests that personal space only exists when interacting with others [4] and hence the term “interpersonal space” is often used as a synonym of “personal space” [5], [6]. Personal space varies across cultures [7], [8], but it is also constantly negotiated according to the context [2], and to the ongoing emotions, which may push us towards or pull us away from others [9]–[11].

With the exponential growth of urban population, city-dwellers often find themselves in situations where they have to pay attention to the presentation of their social self and carefully negotiate their personal space at work, when commuting and also at leisure activities. The introduction of streetcars in the 19^th^ century resulted in the awkward situation of people having to look or be looked at by others for minutes or even hours [12] without any meaningful social interaction (e.g. talking). Anecdotally, SONY corporation developed the first walkman as a means of making the journeys in public transport more tolerable [13]. The ubiquitous use of personal music players in over-crowded public transport alludes to the hypothesis that apart from making the journey more pleasant, listening to music may affect the representation of our own personal space [14], [15] and our interpersonal behaviour.

Overall, music can evoke a wide range of emotional responses in listeners (for a review see [16]). Recent research suggests that the most common goal for people that choose to listen to music is to influence their own emotions, by changing them or releasing them [17]. Hence, the emotional state of the users of personal music players may be often influenced by the music they are listening to; this may intrinsically change their attitude towards other people, and may in turn change their preferred interpersonal distance to the surrounding individuals [2], [10], [14]. Personal music players allow us to take our own music virtually anywhere [14], [15], [18], including public urban spaces, public transport or city streets where strangers surround us and often invade our personal space. Could then be assumed that the use of personal music players triggers specific emotional states that change the personal space boundaries with respect to the strangers surrounding the self?

Recent studies have highlighted the emotional modulation of personal space. For example, it has been found that individual differences in trait feelings of claustrophobic fear relate to the size of personal space [3]. This study provided evidence that people with higher rates of claustrophobic fear show larger peripersonal space, as measured by a line bisection task. Importantly, this study focused on changes on peripersonal space, from a strict cognitive neuroscience perspective, and did not consider how these changes in personal space may in turn affect the interaction with others. In the same vein, a second study suggested that interpersonal distance in humans is regulated by the amygdala [19]. This study reported the case of a patient with complete amygdala lesions that lacked any sense of personal space, as measured by the participant indicating the distance at which she felt most comfortable as the experimenter approached her. Given that the amygdala volume is known to correlate with the human social network size [20], and that the amygdala is known to play a key role in emotion processing, in social approach and avoidance [19] and in the sense of violation of personal space [21], the results of this study seem to indicate a connection between emotion and personal space.

Even though social scientists [14], [15] and psychologists have suggested that “listening to self-selected music may make individuals more comfortable and therefore pay less attention to others in the space around them, thus reducing interpersonal distances” [22], [23], the only empirical study to date [22] has not confirmed this hypothesis. In this study, three different auditory conditions were used: open ears, wearing earplugs or wearing headphones that delivered music described as relatively neutral. Participants were instructed to walk towards the experimenter and to stop when they reached the distance between themselves and the experimenter where they began feeling uncomfortable. Results showed an increase in preferred interpersonal distance in the headphones and earplugs condition, as compared to the open ears condition, thus demonstrating that changes in the auditory cues informing of the space around one's body can influence the preferred interpersonal distance. In addition, four different directions of approach were used (front, back, left or right), with results providing further evidence of a non-spherical shape of personal space, since different dimensions for the different directions of approach to the other person were observed (see also [24]), although no interaction between auditory condition and approach direction was found. However, this study did not explicitly test the relation between personal space and the emotional state of the listeners.

In the present study we aimed to provide the first empirical evidence of the effect of the emotional state induced by listening to music on the personal space of neurologically healthy adults. Specifically, we investigated the effects of the valence and the source of emotion-inducing music on “interpersonal distance”, a term that we use to experimentally operationalize “personal space”, in analogy to other authors considering that personal space only exists when interacting with others [4]–[6]. Our contention is that people find benefit in listening to music through personal music players because, through its emotional regulation, music serves to change the boundaries of their personal space in relation to others located close to them. We hypothesize that the current emotional context determines how the listeners feel against another person invading their personal space: a threatening context may cause one to feel unease and to react against the “invasion” of one's personal space; while in a pleasant context the “invasion” of personal space might be less negative or even positive.

Positive and negative emotion-inducing musical excerpts were used to alter the emotional state of listeners, with respect to a no-music condition. The interpersonal distance was evaluated (see Methods and ref

**Figure 1:**
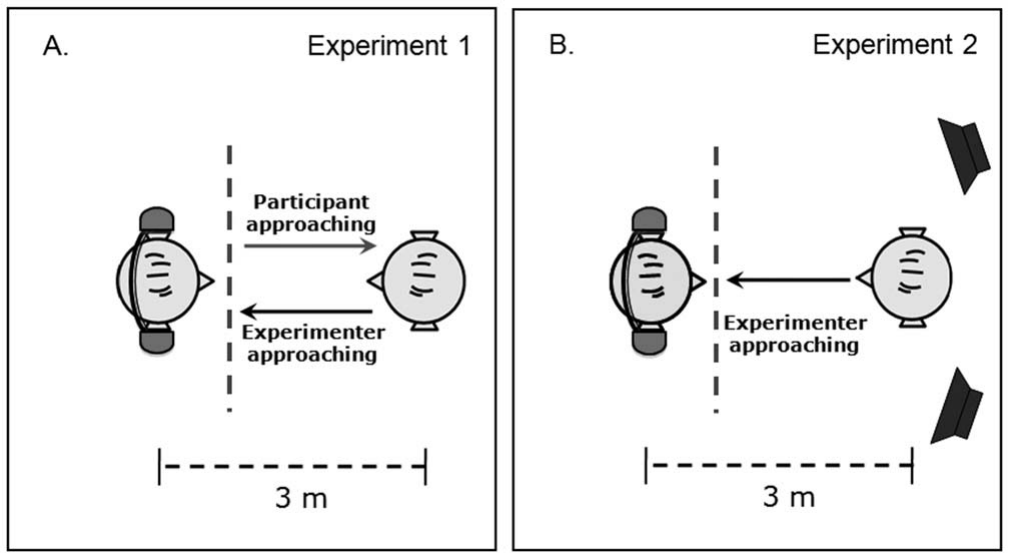
Experimental setup used in Experiment 1 and Experiment 2 (bird's-eye view). (1A) In Experiment 1 auditory stimuli were delivered via headphones. In the approach-distance condition the participant was required to walk towards the experimenter and in the stop-distance condition the experimenter walked towards the participant. (1B) In Experiment 2 in half of the conditions auditory stimuli were delivered via headphones and in the other half via loudspeakers. Experiment 2 only contained conditions with the stop-distance task. Music track and experiment gender were varied across trials.

## Results

The music tracks included in Experiment 1 intended to convey happiness (*positive tracks*) or threat (*negative tracks*). To validate them, we first investigated the effect of the different music tracks on participants' emotional feelings by submitting the self-reported valence and arousal values for the feelings when listening to the different music tracks in a MANOVA containing as within-participants factor ‘music track’ (positive1, positive2, negative1, negative2 and no-music). The results revealed that there was a significant main effect of ‘music track’ (*F*(8, 246) = 27.7, *p*<.001, Λ = .28), which was significant for both valence (*F*(4,124) = 49.8, *p*<.001) and arousal (*F*(4,124) = 14.3, *p*<.001) dimensions. The negative tracks were rated as more negative than their positive counterparts (see Table 1 for means values), thus validating the choice of music tracks in terms of eliciting emotional responses with different valence. It should be noted that in the present study the negative tracks selected elicited a more arousing experience than the positive tracks, although similar arousal values for these tracks were observed in [26].

**Table 1:** Mean effects of the different music in mean valence and arousal emotional ratings (in a 9-point scale) tracks.

	Valence	Arousal
Positive1	7.25 (0.3)	4.19 (0.4)
Positive2	7.84 (0.2)	5.06 (0.4)
Negative1	4 (0.4)	6.97 (0.3)
Negative2	3.4 (0.3)	5.72 (0.3)
No-music	4.37 (0.4)	3.87 (0.3)

Parentheses give the standard errors of the mean.

**Figure 2:**
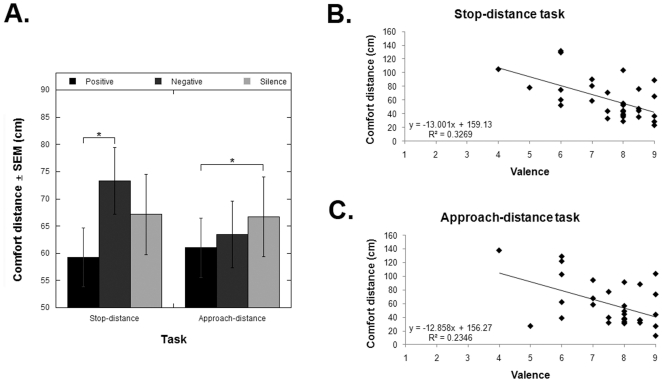
Results of Experiment 1. (2A) Comfort distance ± SEM (cm) for the two different tasks (approach-distance, stop-distance), and for the three different sounds track types (positive, negative, no-music). * mark significance. (2B) Negative correlation between comfort distance in the conditions with positive emotion-inducing music and self-reported emotional valence when listening to this music during the stop-distance task and (2C) the approach-distance task.

We, then, analyzed the behavioural results from the approach-distance and stop-distance tasks. First, we tested whether the distributions of the obtained data were normal using the Shaphiro-Wilk test. None of the factors passed the normality test, therefore we used non-parametrical statistical tests to analyze the data (Wilcoxon Signed Ranks Test). As there were no significant differences between the two tracks used within each valence category (i.e. positive and negative valence), and the gender of the experimenters, we collapsed across these factors. The key observation (see Figure 2A) was a significantly different effect of the two types of emotion-inducing music in the participants' comfort distance during the stop-distance task. For this task, a significantly greater comfort distance was observed for negative music than in the conditions with positive music (*Z* = -3.4, *p*<.001; the critical *p* value after Bonferroni adjustment for multiple comparisons was .017). In Figure 2A it can also be observed a trend to a significant increase in comfort distance resulting from listening to negative music, as compared to the no-music condition (*Z* = -1.75, *p* = .08). A similar disparity between the effects of positive and negative music was not observed for the approach-distance task, in which listening to both negative and positive music when approaching the experimenter resulted in a decrease in comfort distance with respect to the no-music condition, with this decrease being significant for the case of positive music (*Z* = -2.54, *p*<.011). A correlation analysis revealed (see Figure 2B and 2C) a high correlation between the reported emotional feelings induced by the positive tracks and the behavioural results for the conditions involving ‘positive’ tracks for both the stop-distance task (*r* = 0.57, *p*<0.001) and the approach-distance task (*r* = 0.48, *p*<0.005; *p* values corrected for multiple comparisons).

Overall for both tasks, listening to positive music through headphones resulted in a significant change in comfort distance, relative to negative music in the stop-distance task and relative to the no-music condition in the approach-distance task. In particular, the influence of the valence of emotion-inducing music in comfort distance could be observed during the stop-distance task, in which listening to either negative or positive emotion-inducing music, resulted in significantly opposite directions of change in comfort distance to an individual approaching. However, given the use of headphones across all conditions, the current experimental design cannot conclude whether the effects on personal space are due to the valence of music *per se*, or due to the source (i.e. headphones) of the positive music that participants listened to. Experiment 2 was conducted to examine the specific effect in personal space of wearing headphones while listening to music.

**Figure 3:**
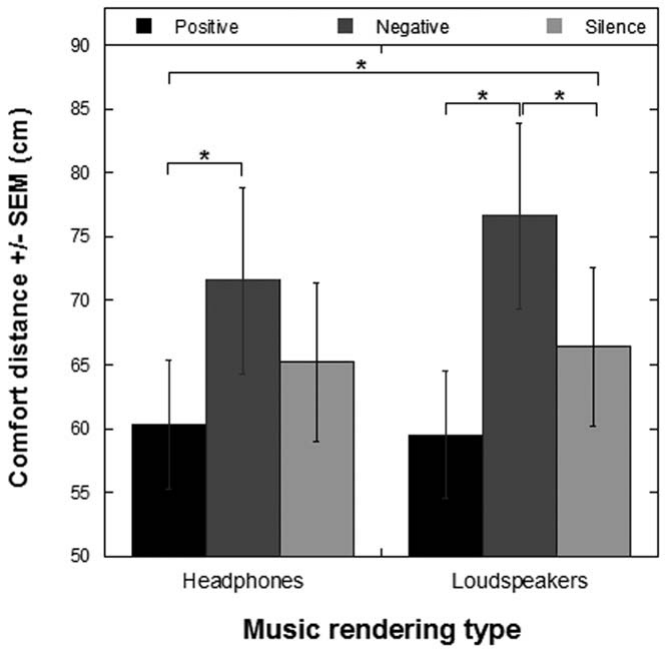
Results of Experiment 2. Comfort distance ± SEM (cm) for the two music rendering types (headphones, loudspeakers), and for the three different sounds track types (positive, negative, no-music). * marks significance.

In Experiment 2, positive and negative emotion-inducing music was delivered either through headphones or through loudspeakers while participants performed the stop-distance task (see Figure 1B). As in Experiment 1, we used non-parametrical statistical tests (Wilcoxon Signed Ranks Test) to analyze the behavioural results since their distributions did not pass the normality tests. Male and female experimenter conditions were averaged across conditions. This experiment replicated the findings of Experiment 1, since a similar pattern of results was observed for the headphones conditions, with a significantly different effect of the two types of emotion-inducing music (i.e. positive versus negative) in the participants' comfort distance (*Z* = -3.2, *p*<.001). However, the novelty of this experiment rests in that we explored the difference between each experimental condition and the no-music loudspeakers (i.e. no headphones) condition. The key observation (see Figure 3) was a significant reduction in comfort distance when listening to positive music through headphones (*Z* = -2.59, *p*<.0096) and a significant increase in comfort distance when listening to negative music through loudspeakers (*Z* = -2.63, *p*<.0086), with respect to the no-music loudspeakers condition (the critical *p* value after Bonferroni adjustment for multiple comparisons was .01). No other significant differences between the other experimental conditions and the no-music condition were observed.

Hence, it can be concluded that it was the combined effect of wearing headphones while listening to positive music that results in a significant reduction of the personal space as compared to the no-music loudspeakers (no headphones) condition. Importantly, the increase of personal space due to negative music was only significant for the loudspeakers condition, as if the origin of negative stimuli, being an external, distant, source to self versus an embedded source, would be a critical factor to impact one's personal space. Both positive music conditions significantly counteracted the effect of the negative music rendered through loudspeakers (*Z* = -3.45, *p*<.0005, for headphones; *Z* = -4.08, *p*<.000046, for loudspeakers).

## Discussion

Listening to emotion-inducing music significantly shifts the margins of personal space in relation to unfamiliar others approaching us. Our results show that when listening to music that induces positive emotions, emanating from an embedded source (music delivered through headphones), our personal space “shrinks”, allowing others to get closer to us. In contrast, our results also show that when listening to music that induces negative emotions, emanating from an external source (music delivered through loudspeakers), our personal space “expands”, resulting in a larger interpersonal distance. Opposite effects of positive and negative emotion-inducing music in personal space were observed for both headphones and loudspeakers listening conditions when the experimenter approached the participant, but not when the participant, wearing headphones, approached the experimenter. This finding seems to indicate that the emotional context does not alter comfort distance to others when one is in control of keeping this distance.

An ample body of research has provided with evidence of the existence of boundaries between one's near, personal space, and the space far away from the body. Neuropsychological, neurophysiological and psychophysiological studies have evidenced that sensory information is processed differently for the space near and far the body, and that there exist brain areas specialized for the processing of sensory information emanating from events occurring in the immediate vicinity of the body [27]–[30]. This specialization of brain areas for the near space has been considered the result of a need for larger visuomotor control in this space [31]–[33], but also of a need for sustaining a margin of safety around one's body, by keeping distance between self and other individuals seen as potential predators [10], [25], [34], [35]. In addition, far and near space are mentally represented differently. For instance, people often show a lateral attentional bias, which shifts from left to right when increasing distance from one's body [36]–[38]. This rightward shift in bias from near to far space is often used to estimate the “size” of near, or personal, space [3]. Recent studies have shown that these representations of personal space are not constant, but that they are continuously updated in response to the current flow of multisensory information; for instance, tool use may result in an expansion of personal space [39]. Similarly, the representations of personal space may also be updated by emotional states; for instance, the feeling of being in a potentially threatening situation may result in an expansion of the personal space [40]. Our results provide empirical demonstration of this relation between the listeners' current emotional state and personal space when interacting with other people. Our findings are supported by previous studies showing that personal space is influenced by claustrophobic fear, which is a pathological emotional state [3], and that the sense of one's personal space is regulated by the amygdala [19], a brain region known for playing a key role in emotion [19], [21].

Importantly, in the present study, changes in the participants' emotional state were induced by music listening, which was not explicitly involved in the task participants were required to perform. Still, the incidental emotional state participants experienced was reflected in the change in comfort distance between participant and experimenter. A high correlation was found between the participants' self-reported emotional state when listening to the positive emotion-inducing music and the behavioral changes in interpersonal distance both when the experimenter approached the participant and when the participant approached the experimenter: the more pleasant the experience of listening to music, the smaller the preferred interpersonal distance between participant and experimenter. This finding is consistent with the notion of emotion signals that suggest that positive emotion signals a safe environment (and hence allow for a smaller personal space) while negative emotion signals an unsafe environment (and thus calls for a larger personal space). A related interpretation comes from the emotion-as-information perspective in social psychology [10]. On this view, experienced emotions provide us with information about objects in our environment, with positive emotions pushing us towards others and negative emotions pulling us away from others [8]–[10], and as such, emotions can shift the boundaries of our personal space: negative emotions seem to pull us away from the individuals invading our personal space, as reflected in the increase of the comfort distance between participant and experimenter; and positive emotions seem to push us towards the individuals invading our personal space, as reflected in the decrease of the comfort distance between participant and experimenter.

Our results also show that the relation between current emotional state and personal space when interacting with other people was influenced by whether the emotion-inducing music emanated from an external, distant, source or from an embedded source (loudspeakers versus headphones listening conditions). With respect to the no-music loudspeakers (no headphones) condition, positive emotion-inducing music was found to reduce significantly the participants' personal space only under the headphones listening condition, while negative music was found to increase significantly the participants' personal space only under the loudspeakers listening condition. These results seem to indicate that positive stimuli emanating from an embedded source and negative stimuli emanating from an external, distant, source have the greatest power to impact one's personal space. These findings are partially supported by previous studies that used close and far sound reproduction techniques (headphones versus loudspeakers) to show a relation between emotional changes and the perceived distance to sound events [41], [42]. It has been reported that listening to news through headphones (versus loudspeakers) seems to shorten the interpersonal distance between the listener and the news anchor, thus providing a more intense, arousing and pleasant experience [41]. Our findings also extend the study by Lloyd et al. [22] on the effect of different auditory input on interpersonal distance by linking changes in the interpersonal behavior of participants with differences on emotional state. In that study, an increase in preferred interpersonal distance during approach-distance tasks when wearing earplugs or headphones with music, as compared to the open ears condition, was found. It should be noted, first, that the earplugs condition is significantly different from a headphones (no-sound) condition, since the former results in a greater elimination of auditory cues, and can induce an awkward feeling in participants, a feeling of alertness which may result in an increase personal space. Second, that in Lloyd's et al. study participants performed an approach-distance task, which, as shown in the present experiment, seems to be less sensitive to the emotion manipulations. And third, that in that study a clear change in the emotional state of listeners was not elicited, since the music played during the headphones condition was described as relatively neutral.

Our study might help to understand the benefit that people find in using personal music players in crowded situations, such as when using the public transport in urban settings. In situations in which there are little possibilities for personal mobility and personal space is constantly compromised, a portable device allowing for a change in the perceived space around would be highly desirable. Some authors have proposed that personal music players and cell phones may have such power to change human perception of the surrounding space [18] and to change the representation of personal space in particular [35]. The present research shows that positive emotion-inducing music listened through headphones contracts personal space, which might help to tolerate crowding more easily. Importantly, the observed contraction of personal space cannot be merely accounted by an increase on attentional load when listening to music, but rather to an effect of the valence of the emotional state induced by the music, since personal space did not contract when listening to music inducing negative emotions, which are known to be more attention-grabbing [43], [44]. However, in order to experimentally extend these results to a real social interaction, and before concluding the effects of personal music players on tolerating crowding, future research should test the paradigm of the current study in a crowded situation, with multiple person interactions and more realistic contexts. Interestingly, it should be noted that research into crowding has found that it is not density per se but the proximity of others that leads to feeling crowded [2]. It might be also interesting to test whether the reported findings on the emotional modulation of the size of the personal space extend to human-machine interactions or if they are restricted to human-human interactions. Finally, a further topic for future research is to investigate more thoroughly how the perceived distance and the perceived location of the emotional auditory stimuli with respect to the listener may affect personal space, since previous research has found that sound objects (or events) perceived in the near space, or heard behind the listener tend to elicit more intense emotional responses in listeners that their counterparts [45], [46].

## Materials and Methods

### Experiment 1

#### Participants

All participants (*N* = 32, 16 female, *M*age = 21, range  = 19–24) had normal hearing, were naive as to the purposes of the study and did not know the experimenters. They gave informed written consent to participate in this study. The study was approved by the Local Ethics Committee, Department of Psychology, Royal Holloway University of London.

#### Apparatus and materials

The experimental room had an approximate size of 70 m^3^ (5.8×4.4×2.7 m). The auditory stimuli consisted of two positive, happy, and two negative, scary, emotion-inducing musical excerpts, hereafter referred as *positive* or *negative* music tracks (Copyright, Bernard Bouchard, 1998; see [26]). For positive music tracks, the mean normative valence and arousal ratings (on a 9-point scale) were, respectively, 6.5 and 8, and for negative music tracks, respectively, 4.4 and 8. *Valence* (or *pleasantness*) and *arousal* (or *activation*) values serve to characterize emotions in a two-dimensional emotional space [47]. Given that the positive and negative tracks were similar in arousal but were placed on opposites sides of the valence axis of the defined emotional space, in the context of the present study happy and scary may be considered opposed on the valence dimension. Hence, in the present paper we treat as synonyms, on one hand the terms “positive” “pleasant” and “happy”, and on the other hand the terms “negative”, “unpleasant”, “threatening” and “scary”.

The musical excerpts, which were composed specifically for the study by Vieillard et al. [26], were instrumental and composed in the genre of film music. These musical excerpts were unknown to the participants, and therefore, no associations based on previous experiences of listening to the selected musical excerpts were expected. All musical excerpts had an approximate duration of 12 s and were repeated twice in one track with a 250 ms beep sound in between repetitions (1 kHz pure tone, 44.1 kHz sampling rate), thus resulting in music tracks with a total duration of approximately 24 s. The sound level was approximately 75 dBA, as measured at the participant's ear position. An additional no-music track, only containing the 250 ms beep at second 13, was created. A 10-ms onset ramp was applied to the auditory stimuli to prevent clipping.

Two pairs of headphones connected to an iPod (Apple®) were used to deliver the auditory stimuli both to the participant and one experimenter.

#### Procedure and design

Participants took part individually in the experiment. During recruitment, they were informed that the study focused on music listening and that we aimed to estimate the distance at which they began to feel uncomfortable either when approaching or when being approached by one of the experimenters. Importantly, no mention to the emotional manipulation performed in the experiment was done.

Two experimenters, male and female, who the participant did not know beforehand, were present during the test, and they were introduced to the participant as “experimenters” (as opposed to fellow subjects). After gaining informed consent, both participant and one of the experimenters (hereafter referred to as Experimenter A) put the headphones on. The participant wore headphones during all the experiment, even during the silent track conditions. The experimenter wore small insert-headphones in order to hear the beep that signaled the beginning of the stop- and approach-distance tasks. It should be noted that the headphones used by the experimenter did not impede him/her to hear the participants' voice. Participants were required to perform the following task. In the beginning of each trial, the participant and Experimenter A stood at opposite sides of the room, along the longest axis crossing the center of the room, and separated by approximately 3 m (see Figure 1A). One of the five possible music tracks was played and the participant listened to it until a short beep occurred, at approximately 12 s after the onset of the music track. This beep signaled the start for the participant to walk towards the Experimenter A (approach-distance task; see [22]), or for Experimenter A to walk towards the participant (stop-distance task; see [2]). The participants' task, which was indicated to them at the beginning of each block and trial, was to stop walking (in the approach-distance task) or to say “stop” (in the stop-distance task) when they began to feel uncomfortable with the distance between them and the experimenter. Then, the participants were asked to close their eyes while the other experimenter (Experimenter B) measured the distance between participant and Experimenter A (chest to chest). It should be noted that participant and experimenter kept the same steady pace of walking through all the experimental trials and that while walking they looked at each other's chin, in order to standardize eye contact [5]. Participants practiced this task prior to the beginning of the experimenter, and both experimenters made sure that the pace was kept constant across conditions. Importantly, all music conditions were chosen to evoke similar arousal in participants, and therefore, no relaxing effect on the participants pace when walking was expected. Stop-distance procedures have been widely used in previous studies (for a review, see [2]), and offer a good reliability (.83; [2]).

Participants completed four different experimental blocks, each including the five different music track conditions presented in random order. The task of the participant (approach-distance or stop-distance) and the gender of Experimenter A were varied across blocks, with the order of the blocks being counterbalanced across participants. This resulted in 20 possible experimental conditions, which differed in the music track presented (two positive valence tracks, two negative valence tracks, and a no-music track), in the gender of the experimenter A (male, female) and in the task of the participant (approach-distance, or stop-distance task).

At the end of the experiment, participants were invited to sit alone next to a desk placed by the wall, and were asked to listen again to all music tracks and rate their emotional feelings using the Self-Assessment Manikin [48], a test widely used in emotion research which consists of two 9-point pictorial scales. One scale serves to rate the valence or pleasantness of emotional feelings, and depicts nine manikins ranging horizontally from happy (or positive) to unhappy (or negative); the other scale, serves to rate the arousal or excitement of emotional feelings, and depicts nine manikins ranging horizontally from excited (or aroused) to calm (or relaxed). On average, the experiment took 20 minutes to be completed.

### Experiment 2

#### Participants

All participants (*N* = 38, 26 female, *M*age  = 24, range  = 18–60) had normal hearing, were naive as to the purposes of the study and did not know the experimenters. They gave informed written consent to participate in this study. The study was approved by the Local Ethics Committee, Department of Psychology, Royal Holloway University of London.

#### Apparatus and materials

A similar apparatus as in Experiment 1, with the exception that in the present experiment the auditory stimuli were delivered in half of the conditions via headphones, and in the other half via a pair of loudspeakers (see Figure 1B). The loudspeakers were identical and were located facing the participant at a distance of 3 m, symmetrically placed 2 m to the right and to the left of the participant, and at a height of approximately 2 m.

The auditory stimuli consisted of three out of the five tracks used in Experiment 1: the no-music track and one music track of each valence type (‘positive2’ and ‘negative2’ were selected, as in Experiment 1 these tracks elicited emotional responses with similar reported arousal; *p*>.28). The sound level for both headphones and loudspeakers conditions was approximately 65 dBA, as measured at the participant's ear position.

#### Procedure and design

The same procedure as in Experiment 1 was followed, with the following exceptions. In this experiment only three music track conditions were used (positive, negative and no-music); the auditory stimuli were delivered either via headphones or via loudspeakers; and only the stop-distance task was used.

Participants completed four different experimental blocks, each containing the three different music track conditions presented in random order. In two of the experimental blocks sound was presented via headphones, while in the other two blocks was presented via loudspeakers. Moreover, in two of the experimental blocks Experimenter A was male and in the remaining two blocks Experimenter A was female. This resulted in 12 possible experimental conditions which differed in the music track presented (positive, negative or no-music), in the gender of the experimenter A (male, female) and in the music rendering type (headphones, loudspeakers). The order of the blocks was randomized across participants. On average, the experiment took 15 minutes to be completed.
