# Correction: I-Space: The Effects of Emotional Valence and Source of Music on Interpersonal Distance

**DOI:** 10.1371/annotation/6a8a7614-6c5d-4d43-ba98-3259c1271493

**Published:** 2012-11-27

**Authors:** Ana Tajadura-Jiménez, Galini Pantelidou, Pawel Rebacz, Daniel Västfjäll, Manos Tsakiris

A portion of the last paragraph in the Introduction section is missing from the online version of the article. It appears correctly in the PDF file. The complete paragraph should read:

Positive and negative emotion-inducing musical excerpts were used to alter the emotional state of listeners, with respect to a nomusic condition. The interpersonal distance was evaluated (see Methods and Figure 1) as the comfort distance on the frontal plane between the participant and an unknown person (the experimenter) during both passive and active approach paradigms. Participant and experimenter faced each other while the experimenter walked across the room towards the participant (‘stop-distance’ procedure; see [2]), or the participant walked towards the experimenter (‘approach-distance’ procedure; see [25]). We focused only in the frontal direction of approach, since previous research has not shown any interaction between auditory condition and approach direction [22], and therefore, the terms ‘‘interpersonal distance’’ and ‘‘interpersonal space’’ are interchangeable in this study. If the ongoing emotional state can actually change the attitude of the listener to the surrounding individuals and alter the perceived need of keeping a margin of safety against potential threats, we hypothesize that positive emotion-inducing music will ‘‘shrink’’ participants’ personal space, while negative emotion-inducing music will ‘‘expand’’ participants’ personal space. In a second experiment we also investigated whether the source of the music, being either an external source (i.e. music played through loudspeakers), or a source ‘‘embedded’’ in the listener’s ears (i.e. music played through headphones), will influence the change on the personal space representation. 

